# A Plea for More Moral Restraint for Students in Our Medical Colleges

**Published:** 1905-11

**Authors:** 


### A PLEA FOR MORE MORAL RESTRAINT FOR STUDENTS IN OUR MEDICAL COLLEGES.

There are three classes of students at medical colleges:

(1) Those who have good morals; are industrious and ambitious, and have a high ideal objective point in life.

(2) Those whose morals are questionable, and are not as industrious as they should be and are lacking in ambition, and consequently their ideal objective point in life is only mediocre. This class of students are easily led into evil habits.

(3) There is a class of students who may have plenty of means furnished them for necessary and other expenses. Their ideal objective point in life is not high. Their morals are corrupt, and they have not been taught the value of economy. This class of students scheme to do evil and endeavor to corrupt others with whom they associate.

These three classes of students being thrown together miscellaneously in boarding-houses and elsewhere without any moral restraint whatever thrown around and about them, it would be very strange if class (3) of students would not lead the majority of class (2) of students into bad habits, and some of class (1) of students.

It is likely that class (1) of students would not be much disturbed in their morals and industry and a high objective point but they would be constantly subjected to temptations by class (3) of students.

Class (2) of students would be in the greatest danger, because they would be more susceptible to temptations from class (3) of students.

There are a great many students of all three classes of students from the country who never have been taught to realize the temptations and dangers of a large city, may be led astray and therefore to do wrong. While class (3) of students would be very certain to form bad habits, class (2) of students would be less liable, and class (1) of students would be least liable.

Let us follow one of these students from the country of class (3) of students to a large city to attend medical college. He goes to a boarding-house without any moral restraint whatever thrown around and about him. If he belongs to class (2) or (3) of students he is soon led into temptations and to do wrong. It may be possible to lead some of class (1) of students, if they are from the country, astray.

The temptations and inducements from class (3) of students are continually having its effects on all the students at medical colleges, and evil communications corrupt good morals, and evil is bound to grow out of the influences of class (3) of students.

The life of many a student has been shipwrecked at medical college by the present mode of housing them in boarding-houses variously located about a large city, without any moral restraint whatever. I can bear witness to the shipwreck of six young men, students at medical colleges, who came under my observation in one city. This has been the experience of every doctor within the hearing of my voice, who has practiced medicine twenty years. Then consider the number of lives of students at medical colleges in the aggregate that have been sacrificed by the present mode of housing them in boarding-houses without any moral restraint whatever.

Provision at every medical college should be made to lodge and board students so that a moral restraint could be thrown about them. Students should be housed and boarded within the walls of every medical college, as they are housed and boarded at most of our literary colleges, so that a supervision in all their actions could be placed over them. Medical colleges should have a head whose duty should be to look after the morals and habits of the students. Until these moral environments are thrown around students, we will continue to reap a full harvest of shipwrecked lives of our students and doctors.

It is the duty of the medical profession to see to it that the morals and habits of every student at every medical college is properly taken care of. The morals and habits of every student when he graduates an M.D., should at least be as good when he takes his diploma home with him, as they were when he matriculated at medical college as a student.

I believe it is the duty of the faculty of every medical college to admit only students with unexceptional habits and morals, and see to it that as long as they are students under their charge that it is their duty to keep a strict surveillance over them, and if they find that any of the students are lapsing into bad habits or morals, they should have them reclaimed, or sent home. Every faculty of every medical college should continually impress upon their students the importance and necessity of each student leading a sober, industrious and moral life.

A young man who came under my immediate observation, after graduating at a literary college, still retaining the good habits the parents taught him at home, matriculated in one of the New York medical colleges; he formed habits which ultimately proved to be his ruination. When he commenced the practice of medicine the habits which he contracted at medical college got the better of him, and he went from bad to worse, till a few years subsequently, he died from the effects of these bad habits. If there had been at the medical college at which he attended, a moral restraint thrown around and about him, this bright and ambitious young man, with a bright future before him, would have turned out differently, and instead of dying prematurely, as he did die, he might have lived to be an honor to himself, to his parents, who loved him, and to all who took an interest in him.

In view of what I have said, and in view of my study and observation of the subject, I am free to state, that no medical college should have a charter that could, and would not, provide for sufficient moral restraint for the students who attend such an institution.

---

### THE PROBLEM OF PREVENTING TUBERCULOSIS.

McFarland regards this as the greatest of all problems, especially since ten millions of the present population are likely to die from the disease, either directly or indirectly. It is a disease common to man and animals, and the sequence of the bacillus of Koch. Whether there are various tubercle bacilli peculiar to different animals is undecided. When an infectious agent passes from man to man in the morbid discharges, in the majority of cases it is from the respiratory apparatus. It may be projected into the atmosphere by cough for several metres and remain suspended a long time. Infection may be derived from animals and from such food products as meat, milk, butter, cheese, etc. Rarely the disease is transmitted from mother to offspring by the placenta; occasionally by coitus when the reproductive organs are diseased. The most frequent avenues are: (1) The skin, by careless contact with infectious material; (2) the respiratory tract into which it may be inhaled; (3) the digestive tract into which it may be swallowed. The first manifestations of the disease are not necessarily in the part of the body into which the bacilli may have been introduced. Preventive measures consist (1) in the protection of the well, (2) in the care of the ill. Instruction of school children, in as simple a manner as possible, concerning the importance of the subject is advised, and adults should be instructed by pamphlets, lectures, newspaper articles, and personal advice from physicians. Suitable legislative measures should be enacted concerning the construction and care of tenements, workshops, public vehicles, and foods. The proper inspection of meat and milk is of the greatest importance. Those who are ill, especially those who remain at home, and are poor and ignorant, should be objects of especial concern and care, on account of others not less than on their own account. Such patients should have done for them by public authorities, in food, clothing, medicine, and in general sanitary care, what they can not do for themselves.—*Medicine, August, 1905.*

